# Crab Shell Extract Induces Prostate Cancer Cell Line (LNcap) Apoptosis and Decreases Nitric Oxide Secretion 

**DOI:** 10.22074/cellj.2016.4879

**Published:** 2017-02-22

**Authors:** Leila Rezakhani, Mohammad Rasool Khazaei, Ali Ghanbari, Mozafar Khazaei

**Affiliations:** 1 Student Research Committee, Kermanshah University of Medical Sciences, Kermanshah, Iran; 2 Fertility and Infertility Research Center, Kermanshah University of Medical Sciences, Kermanshah, Iran

**Keywords:** Apoptosis, Cell survival, Prostate Cancer

## Abstract

**Objective:**

Prostate cancer is the second most common cancer worldwide. Chemotherapeutic agents have been shown to have adverse side-effects, and natural compounds have been recommended for cancer treatment, nowadays. Crab shell has been shown to have cancer preventative and suppressive effects *in vivo* and *in vitro*. The aim of present study was to investigate the effect of crab shell extract on prostate cancer cell line (LNcap) *in vitro*.

**Materials and Methods:**

In this *in vitro* experimental study, LNcap cells were treated with different concentrations (0, 100, 200, 400, 800 and 1000 µg/ml) of crab shell hydroalcoholic extract in three different culture periods (24, 48 and 72 hours). LNcap viability was evaluated by trypan blue staining and MTT assay. Cell apoptosis and nitric oxide (NO) secretion were determined by TUNEL and Griess assays, respectively. Data were analyzed by one-way ANOVA test and P<0.05 was considered significant.

**Results:**

LNcap viability was decreased dose- and time-dependently. Thus 400, 800, and 1000 µg/ml doses showed significant differences compared to control group (P<0.001). Dose-dependent increase in the apoptotic index was also observed in 800 and 1000 µg/ ml concentrations (P<0.001). Nitric oxide secretion of LNcap cell was decreased time- and dose-dependently, while it was significant for 1000 µg/ml (P<0.05).

**Conclusion:**

Crab shell extract showed anti-prostate cancer effect, by inducing cell apop- tosis and decreasing NO production.

## Introduction

Cancer is one of the crucial causes of death worldwide, and prostate cancer is the second most common cancer around the world and the third most common cause of death in developed countries (1). Cancer chemoprevention has been defined as the use of dietary and pharmacological interventions with synthetic agents or specific natural compounds designed to prevent, suppress, or reverse the process of carcinogenesis before development of malignancy (2).

The presence of free radicals cause cell and tissue damage, which is known as oxidative damage (3). Antioxidants are inhibitors of oxidation process and have diverse physiological roles in the body. Antioxidants act as radical scavengers and convert them to less reactive species. A variety of free radical scavenging antioxidants are found in dietary sources like fruits, vegetables and tea (4).

Selenium compounds are active chemopreventive agents. Selenium is a ubiquitous metalloid with properties similar to those of sulfur which have benefits in preventing several types of cancer, including lung, colorectal, head and neck as well as prostate cancers (5). Chemical derivatives of selenium include inorganic compounds such as selenite and selenate, and organic compounds such as selenomethionine (SeMet) and selenocysteine. Different doses, chemical forms and metabolic activity of selenium have anticancer activities (6). The biological activity of Selenium is dependent on its chemical form. Non-organic selenium compounds have shown genotoxic effect, while organic selenium compounds have demonstrated anticancer activity and better toleration (7).

Some potential mechanisms for anticancer effect of selenium have been considered such as antioxidant effect, immune system enhancement, apoptosis induction and cell cycle arrest (8). Studies have suggested that selenium lead to both endothelial and cancer cell reduction in major regulatory molecules of angiogenesis *in vitro*. Selenium reduced angiogenesis in carcinogeninduced rat model (9).

In preliminary reports, people with the lowest blood levels of selenium have been shown to have 3.8 to 5.8 times higher risk of death due to cancer, compared to those who had the highest selenium levels. Besides, patients with prostate cancer have been reported to have lower selenium blood level (10). Nicastro and Dunn (11) showed that selenium had preventive effect and chemopreventive activities on prostate cancer.

There are some remedies in traditional medicine, using crab shell for cancer treatment. Crab shell contains many active components with anticancer and cancer prevention effects such as chitooligosacarides, chitosan, carotenoids and selenium (12, 13). We have previously showed anti-proliferative and apoptosis induction effects of crab shell on breast cancer cell line (MCF7) and human umbilical vein endothelial cell line (HUVEC) (12, 14). Given the importance of prostate cancer and necessity of identification and application of new therapeutic compounds, especially compounds with natural origin, the present study was conducted to determine the effect of hydroalcoholic extract of crab shell on LNcap cell line viability, apoptosis and NO secretion *in vitro*.

## Materials and Methods

In this *in vitro* experimental study, LNcap cell line (Pasture Institute, Iran) was treated with crab shell hydroalcoholic extract (0 μg/ml as control as well as 100, 200, 400, 800, and 1000 μg/ml) in three culture periods of 24, 48, and 72 hours. Each experiment was repeated 3-5 times and the mean of data was analyzed (12, 14). The study was approved by Ethical Committee of Kermanshah University of Medical Sciences (Code 93377, Kermanshah, Iran).

### Extraction

Fresh water crab was prepared and identified in terms of genus and species (Potamon Persicum) by a zoologist (Razi University, Iran). The crab shell powder (5 g) was dissolved in 150 ml of 70% ethanol for 48 hours (15) and then filtered by a filter paper. The powder was dissolved in serumfree RPMI1640 (Gibco) medium and passed through 0.22 μm filter before the final use.

### MTT assay

LNcap cells (15×10^3^) were seeded in each well of 96-well plate and 200 μl RPMI 1640 containing 7% serum was added. After 24 hours incubation, the supernatant of wells were removed and 200 μl of different concentrations of extract (0 μg/ml as control, as well as 100, 200, 400, 800 and 1000 μg/ml) was dissolved in serum-free RPMI1640 medium, added to the wells and incubated again. The cells were incubated in different extract concentrations for 24, 48, and 72 hours. Tetrazolium salt was broken by mitochondrial enzyme succinate dehydrogenase of viable cells, and purple insoluble crystals of formazan were produced in this test.

After incubation, the supernatant in each well was removed and 100 μl of MTT solution (5 mg/ml) was added to each well and incubated for 3 hours. Subsequently, 100 μl Dimethyl sulfoxide (DMSO) was added to dissolve formazan crystals at room temperature for 30 minutes. The optical density (OD) of each well was measured using ELISA reader (STAT FAX 2100, Awareness Technology, Westport, USA) at 570 and 630 nm. The viability of the cells was calculated in each concentration by the following formula:

Cell viability (%)=OD of sample wells/OD of control wells×100 (16).

### Trypan blue assay

A total of 3×10^4^ LNcap cells were seeded in each well of the 24-well culture dish. 750 μl of RPMI1640 medium (containing 10% FBS) was added to each well and kept in CO_2_ incubator for 24 hours. Next, the supernatants were removed and 1 ml of serum-free RPMI1640 medium containing one of the crab shell extract concentrations was added to each well. After 24 hours, the supernatants were removed and were frozen in -20˚C for nitric oxide (NO) measurement. The cells were then detached by trypsin (0.25%, 100 μl) and stained by trypan blue and their viability was calculated. Similarly, the cell viability in different wells was calculated for 48- and 72-hours period.

### TUNEL assay

LNcap cell apoptosis was analyzed by TUNEL (Terminal deoxynucleotidyl transferase dUTP nick end labeling) method which is used to determine DNA fragmentation during apoptosis cell death. LNcap cells were cultured in the 96-well plate with confluency of 15×10^3^ /well. 200 μl of RPMI 1640 medium was added to cells and they were incubated for 24 hours. 200 μl of different doses of crab shell extract was then added after removing supernatant, and the cells were incubated for 72 hours. The cells were subsequently fixed in paraformaldehyde (4%) for 1 hour. Penetrability of the cell membrane was increased by ice-cold 0.2% Triton x-100 solution (Sigma, USA) for 2 minutes. The cell was then incubated by TUNEL solution at the temperature of 37˚C and dark condition for 1 hour. Finally, the differential staining of the cell was performed in 5 μg/ml propidium iodide (PI) and the cells were analyzed by fluorescence microscope (Eclipse TS100, Nikon, Japan) after three times washing with PBS. The apoptotic index of the cells was calculated as the percentage of apoptotic cells relative to the total cell number (17).

Apoptotic index (%)=(number of apoptotic cells/total number of cells)×100

In this part, 10 microscopic fields of each well were examined randomly (Magnification of 200x). LNcap cells treated with 10% ethanol (a potent inductor of apoptosis), for 2 minutes, were considered as positive control.

### Nitric oxide assay

NO was measured by Griess staining method. 400 μl of supernatant of each sample was deproteinized by adding 6 mg zinc sulfate. Samples were centrifuged in 4˚C temperature and 12000 g for 12 minutes. 100 μl of 0, 6.25, 12.5, 25, 50, 100 and 200 μM sodium nitrite was added to wells as standard, and 100 μl of the surface liquid of deproteinized sample was added to the other wells. 100 μl vanadium chloride, 50 μl sulfanilamide and 50 μl N-(1-Naphthyl) ethylenediamine dihydrochloride (NEDD) were added to each well. The wells were incubated for 15 minutes and they were then read by ELISA Reader (STAT Fax100) with wave lengths of 540 and 630 nm (18).

### Data analysis

Data were expressed as mean ± SD and analyzed by SPSS16 software using one-way ANOVA test. P<0.05 was considered statistically significant.

## Results

### LNcap viability

**Fig.1:**
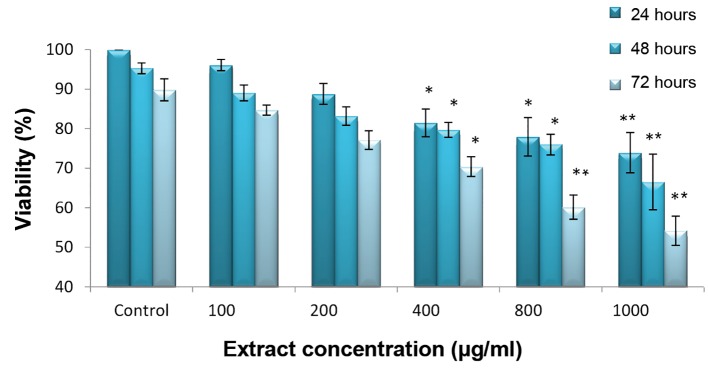
LNcap cell viability (%) level measured by MTT assay after 24, 48 and 72 hours treatment with different concentration of crab shell extract. 400, 800 and 1000 μg/ml concentrations showed significant differences, compared to control group. *; P<0.05 and **; P<0.001.

**Fig.2:**
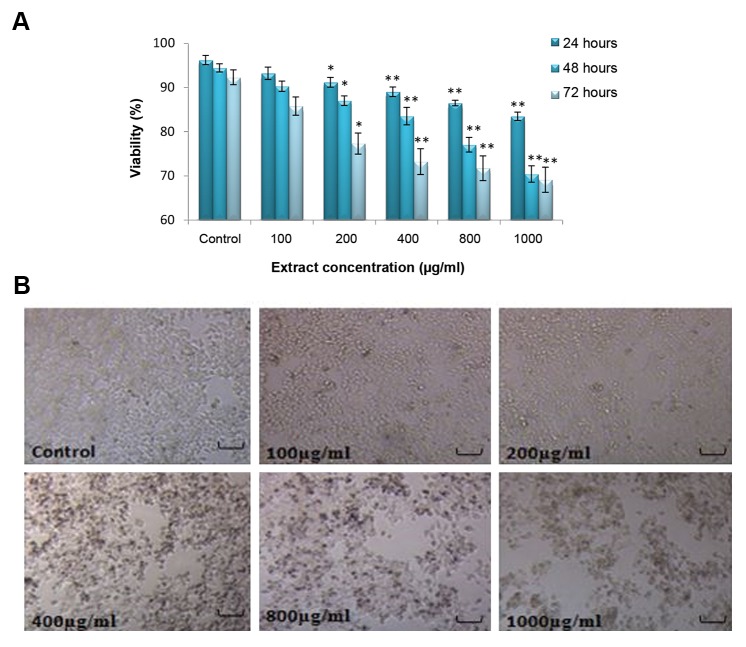
Viability and morphology of LNcap cells, A. LNcap cell viability (%) level was determined by trypan blue after 24, 48 and 72 hours exposure to different doses of crab shell extract. Cell viability was significantly reduced by the time in 200, 400, 800 and 1000 μg/ml concentrations, in comparison with control and B. LNcap cell confluency in different doses of the extract at 72 hours (magnification ×100, scale bar=1 μm). *; P<0.05 and **; P<0.001.

MTT test showed a significant difference between the viability of groups treated with crab shell hydroalcoholic extract (400, 800 and 1000 μg/ml) and control group in 24, 48, and 72 hour periods. The viability of cells was significantly decreased by increasing the extract dose (Fig .1, Table 1). In addition, LNcap cell viability analyzed by trypan blue method in 24, 48, and 72 hour periods indicated a significant difference between treated groups with crab shell extract (200, 400, 800 and 1000 μg/ml) and control (P<0.001, Fig .2A, B).

### LNcap apoptosis

**Fig.3:**
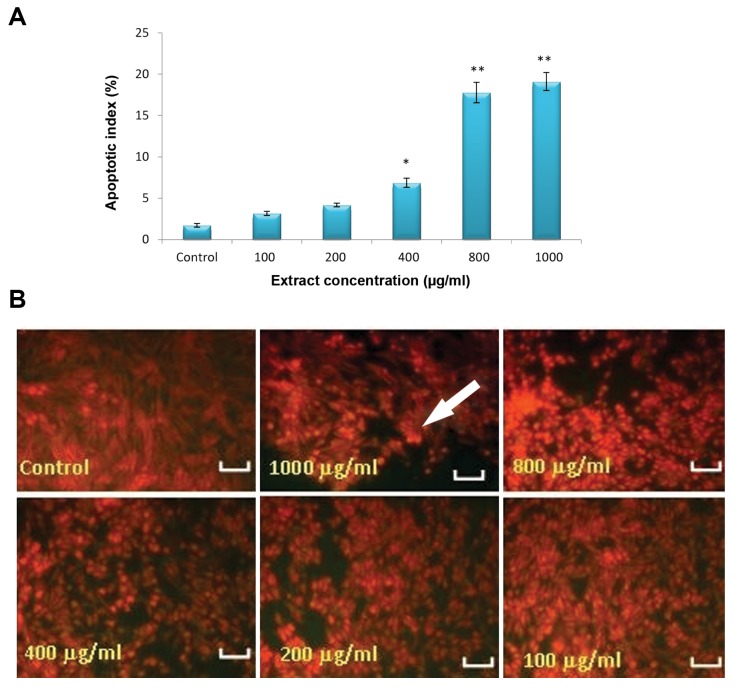
Apoptosis of LNcap cells, A. Apoptotic index (%) of LNcap cell after 72 hours exposure to different concentrations of crab shell extract. 400, 800 and 1000 μg/ml showed significant differences, in comparison with control group and B. Apoptotic LNcap cell in different groups after 72 hours (magnification: ×200, scale bar=1 μm). *; P<0.05 and **; P<0.001.

Apoptosis index of LNcap cells treated with crab shell extract showed significant increase in 400, 800 and 1000 μg/ml, compared to control (Fig .3A, B).

**Table 1:** MTT method. Comparisons of viability for cancer cell lines LNcap after treatment with different doses of crab shell extract at 24, 48 and 72 hours by MTT method


Group (µg/ml)	24 hours	48 hours	72 hours

Control	100 ± 0	100 ± 0	100 ± 0
100	96.08 ± 1.44	89.03 ± 2.00	84.67 ± 1.28
200	88.77 ± 2.64	83.20 ± 2.31	77.08 ± 2.37
400	81.47 ± 3.48^*^	79.71 ± 1.89^*^	70.37 ± 2.50^*^
800	77.95 ± 4.87^*^	75.97 ± 2.62^*^	60.14 ± 3.05^*^^*^
1000	73.90 ± 5.09^*^^*^	66.50 ± 7.04^*^^*^	54.16 ± 3.71^*^^*^


Differences in concentrations of 400, 800 and 1000 μg/ml are significant. *; P<0.05 and **; P<0.001.

### Nitric oxide levels

NO concentration was evaluated by Griess method. The effect of different concentrations of crab shell extract on LNcap cell line in 24, 48, and 72 hour periods indicated reduction of NO secretion in a dose- and time-dependent manner. This difference was significant for the dose of 1000 μg/ ml, compared to control group (Table 2).

### Extract compounds

Using atomic absorption-VGA mode machine, we determined that the level of selenium was 12.5 ppm.

**Table 2:** Nitric oxide (NO) levels in the culture media of LNcap cell in different concentrations of crab shell extract at 24, 48 and 72 hours


Group (µg/ml)	24 hours	48 hours	72 hours

Control	80.79 ± 8.90	73.85 ± 5.41	70.42 ± 5.57
100	70.40 ± 8.18	65.85 ± 5.16	63.42 ± 3.85
200	67.70 ± 13.26	63.02 ± 4.48	62.38 ± 5.89
400	59.87 ± 5.16	57.33 ± 1.10	57.19 ± 4.93
800	56.35 ± 5.77	55.70 ± 3.55	52.53 ± 8.42
1000	52.92 ± 9.90	52.37 ± 2.67	47.81 ± 2.42*


Differences in 1000 μg/ml concentration are significant. *; P<0.05.

## Discussion

In this *in vitro* study, the effect of crab shell hydroalcoholic extract on LNcap cell line was evaluated. The effects of six doses of extract (0, 100, 200, 400, 800, and 1000 μg/ml) were analyzed in three periods of time (24, 48, and 72 hours). To determine the cells’ viability, MTT assay and trypan blue methods were applied. The highest decrease in cell viability (to 50%) was observed in the 72 hours period with 1000 μg/ ml extract concentration. To determine significant decrease in the cell viability, the 72 hours period was used for apoptosis analysis. Apoptosis indicated a rising trend by increasing the extract dose and a significant increase was observed in 400, 800 and 1000 μg/ml doses. Moreover, NO level was assessed after LNcap treatment with extract, during 24, 48, and 72 hours. NO secretion was significantly decreased depending on the dose and time.

Crab shell extract showed apoptotic effect on LNcap cell and decreased cell viability to 54% at 72 hours treatment, in this study. This extract also decreased the viability of MCF7 and HUVEC to 50 and 63%, with similar dosages, in our previous studies (12, 14). The apoptotic index was 19, 21 and 8% for LNcap, MCF7 and HUVEC cells, respectively. It can be concluded that the extract showed stronger anti-proliferative effect on cancer cells than the normal cell line (HUVEC). Furthermore, crab shell extract showed more potential effect on MCF7, rather than LNcap in terms of viability and apoptosis.

NO secretion levels by LNcap, MCF7 cells and HUVEC were decreased to 47, 30 and 18 μM, respectively in 1000 μg/ml concentration after 72 hours treatment (12, 14). These studies and our finding showed a further reduction of NO was released in cancer cells, compared to normal cells.

The other study revealed that the anti-proliferative properties of fresh water crab shell extract was attributed to the presence of carotenoids, chitin derivatives (chitooligosaccharide and chitosan) and selenium through inducing apoptosis and decreasing NO secretion (14). In another study, the effect of selenium compound was tested on different cancer cell lines. Selenite, methylselenocysteine (MSC) and SeMet were examined on the three cancer cell lines: HSC-3, HSC-4 (carcinoma) and A549 (lung adenocarcinoma). Apoptosis induction of selenium compounds in oral carcinoma was performed by activation of caspases 3, 8 as well as 9, and through p53 pathways in the lung cancer cell line. Apoptosis in both cell lines (19) was similar to our study. Due to the presence of selenium in the crab shell extract, induction of apoptosis in LNcap cell line could be attributed to this compound.

There are other studies, investigated the impact of different selenium compounds on various cancer cell lines, including selenic acid (MSA) which inhibits various breast cancer cell lines (MDA-MB-468 and MCF-7) and prostate cancer cell line (DU145) by reducing vascular endothelial growth factor (VEGF) (20). Therefore, owing to the high selenium content of crab shell extract, one of the probable mechanisms is VEGF reduction in prostate cancer cell line.

## Conclusion

Crab shell extract inhibits the proliferation of prostate cancer cell line in a dose- and time-dependent manner. It seems that crab shell exerts can affect via apoptosis induction and NO reduction. In this line, selenium is considered as a factor in the process of growth inhibition.
